# Extending electronic medical records vector models with knowledge graphs to improve hospitalization prediction

**DOI:** 10.1186/s13326-022-00261-9

**Published:** 2022-02-22

**Authors:** Raphaël Gazzotti, Catherine Faron, Fabien Gandon, Virginie Lacroix-Hugues, David Darmon

**Affiliations:** 1 Université Côte d’Azur, Inria, CNRS, I3S, 2004, route des Lucioles, Sophia-Antipolis, BP 93 06902 France; 2 Université Côte d’Azur, RETINES, Département de Médecine Générale, 28, Avenue de Valombrose, Nice, 06107 France

**Keywords:** Electronic medical records, Predictive model, Knowledge graph, Feature selection

## Abstract

**Background:**

Artificial intelligence methods applied to electronic medical records (EMRs) hold the potential to help physicians save time by sharpening their analysis and decisions, thereby improving the health of patients. On the one hand, machine learning algorithms have proven their effectiveness in extracting information and exploiting knowledge extracted from data. On the other hand, knowledge graphs capture human knowledge by relying on conceptual schemas and formalization and supporting reasoning. Leveraging knowledge graphs that are legion in the medical field, it is possible to pre-process and enrich data representation used by machine learning algorithms. Medical data standardization is an opportunity to jointly exploit the richness of knowledge graphs and the capabilities of machine learning algorithms.

**Methods:**

We propose to address the problem of hospitalization prediction for patients with an approach that enriches vector representation of EMRs with information extracted from different knowledge graphs before learning and predicting. In addition, we performed an automatic selection of features resulting from knowledge graphs to distinguish noisy ones from those that can benefit the decision making. We report the results of our experiments on the PRIMEGE PACA database that contains more than 600,000 consultations carried out by 17 general practitioners (GPs).

**Results:**

A statistical evaluation shows that our proposed approach improves hospitalization prediction. More precisely, injecting features extracted from cross-domain knowledge graphs in the vector representation of EMRs given as input to the prediction algorithm significantly increases the F1 score of the prediction.

**Conclusions:**

By injecting knowledge from recognized reference sources into the representation of EMRs, it is possible to significantly improve the prediction of medical events. Future work would be to evaluate the impact of a feature selection step coupled with a combination of features extracted from several knowledge graphs. A possible avenue is to study more hierarchical levels and properties related to concepts, as well as to integrate more semantic annotators to exploit unstructured data.

## Introduction

Patients are accustomed to meet with their general practitioners (GPs) for their health problems and as such, the electronic medical records (EMRs) in the GP’s possession are along the best available data sources for understanding factors related to the patient’s health condition. These records concern everyone, and each patient is unique, with regard to the biometric information (age, weight, gender...), diseases, interventions and lifestyle behavior. Medical records therefore represent a tremendous opportunity for the development of applications in the field of artificial intelligence to improve patient care. Our case study focuses on the prediction of hospitalization, a scenario motivated by general practitioners who have difficulties prescribing for comorbid patients, a condition that is becoming more widespread due to the overall aging of the population.

We propose to enrich private data (EMRs) with public data (bio-medical knowledge graphs) available in standard Web format (semantic Web and linked data frameworks). Our decision making support system then relies on machine learning approaches trained and predicting on the enriched representations. The combination of these two artificial intelligence techniques is evaluated by measuring the quality of the decision support for deciding an hospitalization.

On the one hand, machine learning algorithms have proven their effectiveness in extracting information and exploiting the knowledge extracted from data on which they are trained but it may be complex for them to rely solely on unstructured or weakly structured information, either because of a context with few data or because some correlations may be difficult to establish with weakly structured data. On the other hand, there are knowledge graphs that organize information based on conceptual schemata and are used to integrate and reason on semantically enriched data. We identified these knowledge graphs as an opportunity to enrich the vector representations used by machine learning algorithms. However, knowledge graphs contain a lot of information that is not suited to a goal-oriented task, which may lead to lesser results by the introduction of noise. The selection of this knowledge could be legitimately left to experts, but like any annotation task, finding an agreement on what is relevant to arrive at a diagnosis is complex for humans, especially when the decision to hospitalize a patient involves many factors.

In this paper, we tackle the general research question *Which contribution from knowledge graphs can improve the prediction of the occurrence of an event?* and, considering the case study on predicting a patient’s hospitalization, we aim to answer the following sub-questions:

- *Which representation and machine learning algorithms are best suited for predicting hospitalization and interpreting the algorithm’s decisions over heterogeneous data?*
- *Do ontological augmentations of the features improve the prediction of the occurrence of an event?*
- *Which knowledge should we extract and select for the prediction of the occurrence of an event?*

In addition, the issue of explainability has been considered from the early stages of the project i.e., from the choice of the machine learning algorithms and the vector representation to the use of knowledge graphs which provide reasoning capabilities.

We evaluated our proposed approach on a dataset extracted from the PRIMEGE PACA relational database [1], which contains more than 600,000 consultations in French, collected from the consultation software of 17 general practitioners. Table 1 specifies the fields of PRIMEGE and Table 2 the volume of data collected.

**Table 1:** Data collected in the PRIMEGE database

Category	Data collected
GPs	Sex, birth year, city, postcode
Patients	Sex, birth year, city, postcode
	Socio-professional category, occupation
	Number of children, family status
	Long term condition -LTC- (Y/N)
	Personal history
	Family history
	Risk factors
	Allergies
Consultations	Date
	Reasons of consultation
	Symptoms related by the patient
	and medical observation
	Further investigations
	Diagnoses
	Drugs prescribed (dose, number of
	boxes, reasons of the prescription)
	Paramedical prescriptions
	Medical procedures

**Table 2:** Data volume in the PRIMEGE database

Element	Amount
Patients	68,415
Consultations	601,464
Past medical history	212,797
Biometric data	384,087
Reasons of consultation	345,626
Diagnoses	125,864
Prescribed drugs	1,089,470
Symptoms	33,273
Health care procedures	15,001
Additional examination	1,281,300
Paramedical prescription	25,910
Observations/notes	73,336

The paper is structured as follows. First, we introduce with motivating scenarios the reasons that led us to formulate our research questions and we discuss the related work. Then we compare and evaluate a sequential and non sequential representation of EMRs to determine which one to adopt as a basis for semantic enrichment. Then, we present and evaluate the methodology we followed to select knowledge and inject it in a vector representation of EMRs. Finally, we conclude and present some perspectives.

## Motivating scenarios: the health predict project and application

In the context of the Health Predict project, we aim at preventing the hospitalization of patients or at least at improving their health’s condition, whether physical or mental, by prioritizing the different risk factors responsible for the hospitalization. The results of this research are intended to provide decision support tools for general practitioners to assist them in their daily practice. Ordering by priority the hospitalization risk factors to be treated is a key issue to support GPs in identifying the best treatment plan, as well as to take into account polypathologies, meaning dealing with drug interactions, and to get the patient’s adherence to his or her treatment.

We present two motivating scenarios designed from the medical record of a real patient and which show the needs of both the physician and the patient. Indeed, both the patient and the GP are committed to preserving or improving the patient’s autonomy and avoiding hospitalization. The GP also wants to be able to predict his patient’s hospitalization as quickly and easily as possible. The HealthPredict application was designed from the beginning with the idea of providing personalized views to both the GP and the patient. The GP’s view provides the current and forecasted risk of hospitalization for a patient after treating his pathologies identified as hospitalization risk factors. The patient’s view wants to facilitate the patient’s therapeutic compliance and thus only shows him the total gain on his hospitalization risk if he complies with the treatment recommended by his GP.

### Scenario 1 - Dr. Nathalie predicting the hospitalization of her patient Patrick (57 y.o.)

**Fig. 1:**
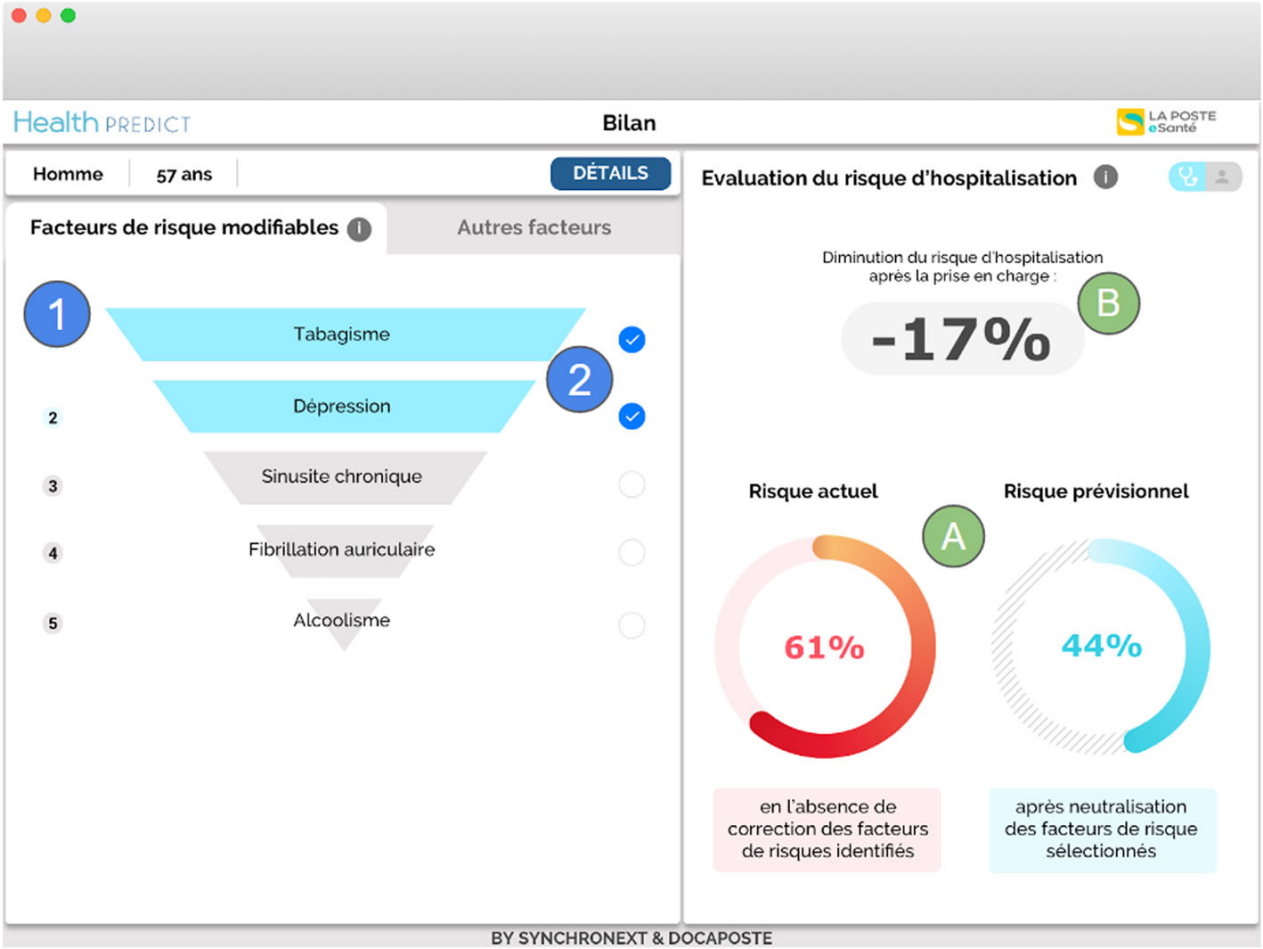
GP view on factors strongly involved in predicting a patient’s hospitalization and on which the GP can intervene. The right part of the window shows the reduction of the hospitalization risk (marks A and B) when the risk factors selected in the left part are managed. The selected risks (marks 1 and 2) are in Blue and are ‘Smoking’ and ‘Depression’. The other risk factors on which the doctor can intervene are in Grey: chronic sinusitis, atrial fibrillation and alcoholism

Once connected to Health Predict through a plugin directly integrated into her consultation software, Dr. Nathalie is considering whether she should hospitalize her patient. Figures 1 and 2 show her Health Predict interface.

1. She checks the five hospitalization risk factors of Patrick (smoking, depression, chronic sinusitis, atrial fibrillation, alcoholism) that she can act upon to reduce the hospitalization probability (in Fig. 1).

**Fig. 2:**
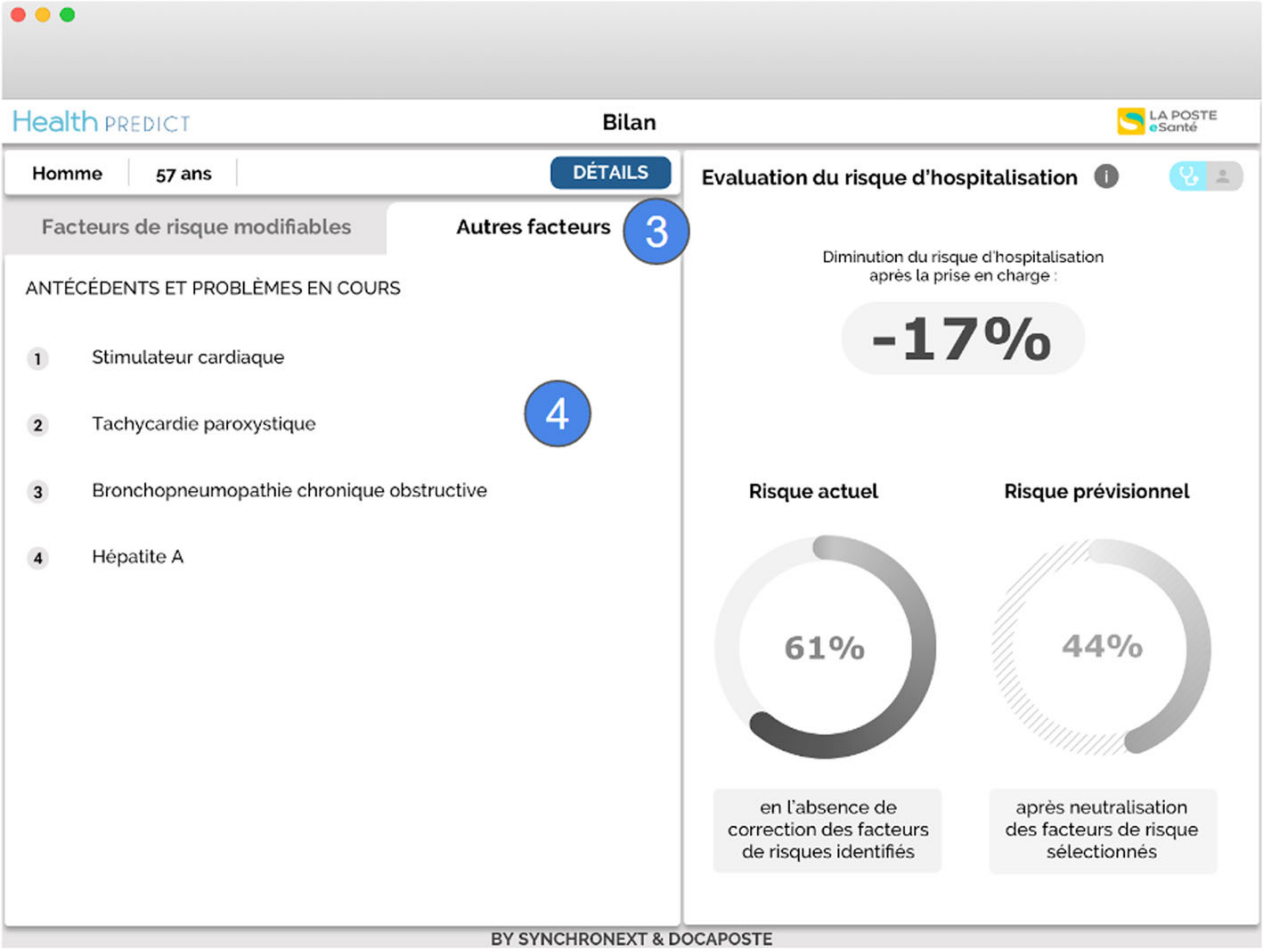
GP view on factors that have a lesser impact or on which the GP cannot intervene (marks 3 and 4). These factors are: cardiac pacemaker, paroxysmal tachycardia, chronic obstructive bronchopneumonia, and hepatitis A
2. She selects the first two risk factors (those with the most impact, i.e., ‘Smoking’ and ‘Depression’) for a treatment. In area (A), she observes the estimated decrease in the risk of hospitalization resulting from the selected risk factors to be treated: it goes from 61% to 44%; in area (B) she sees the degree of this decrease in risk – 17% (Fig. 1).
3. Dr. Nathalie verifies the other risks of Patrick factors to avoid side effects or contraindicated treatments (Fig. 2).
4. She identifies that some antidepressant can be contraindicated with the patient’s condition, since most antidepressants have side effects on the cardiovascular system [2]; the risks include: atrial fibrillation (Fig. 1), cardiac pacemaker (Fig. 2), paroxysmal tachycardia (Fig. 2). Other risks factors are also listed in Fig. 2: chronic obstructive bronchopneumonia, hepatitis A.

### Scenario 2- patient Patrick (57 y.o.) negotiating the treatment to prevent his hospitalization with Dr. Nathalie

**Fig. 3:**
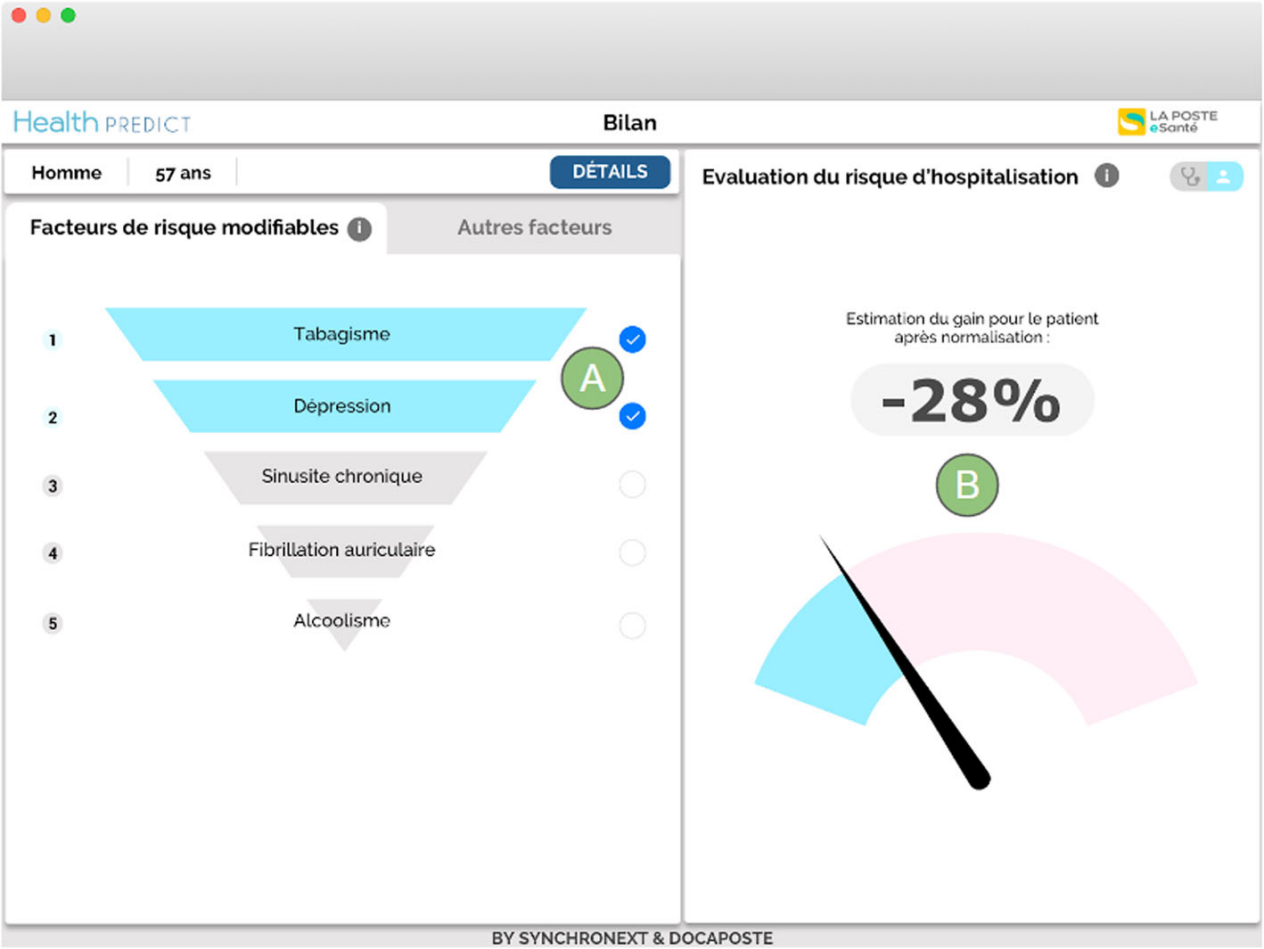
GP view on factors that have a lesser impact or on which the GP cannot intervene (marks 3 and 4). These factors are: cardiac pacemaker, paroxysmal tachycardia, chronic obstructive bronchopneumonia, and hepatitis A

Dr. Nathalie wants her patient Patrick to stop smoking and drinking. She also plans to deal with Patrick’s depression (Fig. 3). Patrick does not feel ready to quit smoking and drinking at the same time. She shows the patient view of her consultation application to Patrick. He can see that stopping smoking and treating his depression would make him 28% less likely of being hospitalized (in Fig. 3). This total gain is computed as the ratio between the hospitalization risks with and without treating the selected risks (61% and 44% in Fig. 1). It is displayed in the patient view to give a less anxiety-provoking message than the one in the GP view where the hospitalization risk values with or without treating the selected risks are displayed.

While studying together the patient view of the consultation software, Dr. Nathalie and Patrick negotiate and agree that Patrick will make an effort to quit smoking and will be assisted by a professional to treat his depression (in Fig. 3). He will also talk about his alcohol problem with this same professional, but the withdrawal of this addiction will have to be smooth.

## Related work

With the final aim to create a tool for general practitioners like presented in the previous section, we were interested in the contribution of knowledge graphs in the prediction of a patient’s hospitalization. In the following, we provide an overview of previous works focusing on using knowledge graphs to contribute to the improvement of machine learning algorithms applied to the biomedical domain.

In [3], the authors aim to discover rules on the daily activities of cancer patients and achieve better performances in the coverage of inferred rules and their interpretations by using ‘IS-A’ relations extracted from the Unified Medical Language System (UMLS)^1^. They exploit the full sub-hierarchy of kinship and co-hyponymous concepts of the OWL representation of UMLS with a machine learning approach to improve the coverage of discovered rules. In addition to the fact that their work is focused on a different purpose than ours, they also did not consider other relations than ‘IS-A’ relations, and they rely only on the AQ21 algorithm and the extension of this algorithm AQ21-OG to study the impact of this enrichment.

In [4], the authors aim at overcoming data insufficiency and to provide a better interpretation of neural networks on the prediction of rarely observed disease. They fed an attention graph-based neural network with ancestors extracted from ICD Disease, Clinical Classification Software (CCS) and Systematized Nomenclature of Clinical Terms in Medicine (SNOMED-CT). These knowledge graphs are transformed using the embedding obtained with Glove [5] to be processed by attention mechanism. This setup outperforms a standard recurrent neural network to identify rarely observed pathologies in training samples. Also it generalizes better with few training data. The smallest dataset used in this study, MIMIC-III, is 5 times larger than ours and their neural network approach is not suited to the amount of data in PRIMEGE. Their approach only considers the ancestors (super class relations) of different biomedical ontologies. Moreover they use attention mechanisms while we evaluate a feature selection approach.

In [6], the authors achieve better classification results than other state-of-the-art approaches using deep learning with a new deep learning architecture based on transformers called Mutual Integration of patient journey and Medical Ontology (MIMO) that they applied on the MIMIC-III and eICU datasets. This approach reuses the same graph embedding approach as in [4] but this time only with the Clinical Classification Software (CCS) and therefore suffers from the same flaws, i.e., taking only into account the ancestors.

In [7], no matter the classifier used, the authors improve in various natural language processing tasks such as information retrieval, information extraction and text summarizing by combining bag-of-words (BOW), biomedical entities and UMLS. We studied a similar outcome but with different knowledge graphs, both general and specific, and we proposed a semi-supervised approach to select knowledge relevant for the hospitalization prediction task.

In this paper we summarize and integrate the works we carried out on integrating knowledge from various knowledge graphs [8], and on the extraction of relevant concepts from DBpedia [9] to predict hospitalization from EMRs. These works have notably led to the publication and defense of a PhD thesis [10]. We also go beyond these initial results and we present in this paper the evaluations that led us to opt for non-sequential algorithms and we confirm our early results by means of a statistical test and by comparing more precisely the F1 results and their standard deviation. In particular we provide a detailed account of several metrics for the best approach against the different augmentation alternatives.

The first step in predicting from EMRs is to determine the representation that will support both the prediction and its interpretation.

## Methods

### Predicting hospitalization from text-based representations of electronic medical records

Our prediction task can be defined as follows: Let *R* be a representation of an EMR from the PRIMEGE Database *P*. Let *C* be the set of classes to predict *C*={*Hospitalized*,¬*Hospitalized*}. We learn the mapping *M*: *M*(*R*)=*L*, where *M* is a classification algorithm that predicts a class *L*∈*C* for an EMR *R*.

Before we can consider the enrichment of an EMR representation *R* with ontological knowledge, the first question to be answered is to determine which EMR representation is best suited to predict a patient’s hospitalization. Since EMRs are essentially based on text data (i.e., the observation field, personal history, family history, etc.), we considered text-based representations. Another important focus with regard to text representations is to retain control over the interpretability of the decisions made by the machine learning algorithms used so that they can be justified and presented to the referring physicians.

#### Vector models of text data in electronic medical records

EMRs present in the PRIMEGE corpus contain a highly specialized terminology in French with abbreviations, which means that the vocabulary used is adapted to general medicine with sometimes references to specialists who may have been consulted by the patient. This led us to adopt our own vector representation and in particular, we use a bag-of-words (BOW) representation to avoid a lack and misuse of specialized terms from which other approaches (e.g., word embeddings) suffer. This representation has the advantage that it does not require a large amount of data and allows to identify the contribution of the features in the hospitalization (or not) of a patient. More advanced representation models experience a loss of information (by compressing the training data), they may also require a larger corpus, and we were concerned to provide GPs with the closest possible details of their patient records as feedback.

*Temporal models of electronic medical records.* There is a great deal of variability in the patient-physician relationship, with some people seeing their doctors on a regular basis over many years and others coming to see them only occasionally. In order to take this temporal dimension into consideration, medical records can be studied under two representations, a sequential representation and a non-sequential representation, that we compared.

We evaluated the alternatives on a balanced dataset *DS*_*B*_ containing 714 patients hospitalized and 732 patients who were not hospitalized over a 4-year period. These data are from between 2012 and 2015, therefore before the SARS-Cov2 pandemic. This detail is important because the recent pandemic introduces a major bias that would require modifying the models by adding hospitalization weighting factors, or otherwise address this particular issue.

*Non sequential modelling of electronic medical records.* The PRIMEGE database is structured with different text fields, so we introduced prefixes in the creation of the bag-of-words to track the respective contributions. Thus, it is possible to trace the fields used to generate the features and to distinguish them in the vector representation of EMRs, e.g., a patient’s personal history vs. his family history.

Our non sequential representation of EMR is as follows. Let $V^{i}=\left \{w_{1}^{i},w_{2}^{i},..., w_{n}^{i}\right \}$ be the bag-of-words obtained from the textual data in the EMR of the *i*^*th*^ patient. To consider this non sequential representation, we had to aggregate all the consultations occurring before a hospitalization. For patients who have not been hospitalized, all their consultations are aggregated. On the one hand, it contains consultation notes on the reasons for the consultation, diagnoses, prescribed drugs, observations. On the other hand, it contains textual information conveyed throughout the patient’s life including, for instance, familial history, personal history, personal information, past problems, the environmental factors as well as allergies. We are in the presence of two classes, thus the labels *y*_*i*_ associated with *V*^*i*^ used for this representation are either ‘hospitalized’ or ‘not hospitalized’.

*Sequential modelling of electronic medical records.* For a sequential modelling of EMRs, we chose to represent the different consultations of a patient as a sequence (*t*_1_,...,*t*_*n*_). This n-tuple contains all his consultations in chronological order, with *t*_1_ his first consultation and *t*_*n*_, his last consultation present in the database. Each consultation *t*_*i*_ contains both persistent patient data and data specific to the *i*^*th*^ consultation. Similarly to the non sequential representation of EMRs, for patients who have not been hospitalized, all their consultations are integrated in the sequential representation of EMRs whereas for patients who have been hospitalized only their consultations occurring before hospitalization are integrated.

**Fig. 4:**
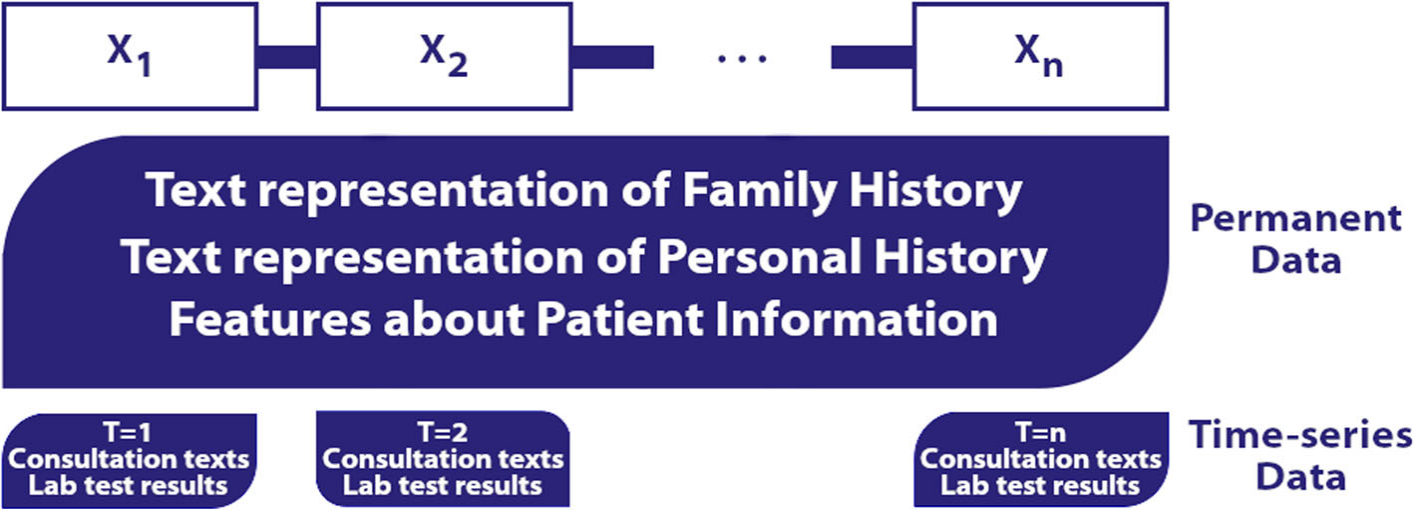
Diagram illustrating the sequential representation of an electronic medical record

Thus *t*_*i*_=(*x*_*i*_,*y*_*i*_) where *x*_*i*_ contains two broad types of information about the patient, general information about the patient and information obtained during a consultation, as described in the section about non sequential modelling of EMRs, the Fig. 4 shows how this data is handled in this representation.

Textual information carried throughout the patient’s life is thus repeated across all *x*_*i*_ of *t*_*i*_.

#### Selected machine learning algorithms

For non sequential classification algorithms, we focus on three different machine learning algorithms which are frequently used in the literature: the logistic regression (LR) [11], random forests (RF) [12], and support vector machine (SVM) [13]. These algorithms, in particular logistic regression and random forests are widely used in the prediction of risk factors from EMR [14]. Moreover, they are natively interpretable in their decision: they provide both the features that are involved in a prediction and the weights learned for the features in a vector representation, except for SVMs where this is the case only for models with a linear kernel.

Markovian models are sequential machine learning algorithms that share the particularity of being interpretable since it is possible to obtain the weights of the state and transition features. Among them, Hidden Markov models (HMMs) are generative models, so they assume that the features are independent, which is not our case with EMRs (e.g., drug interactions, relations drugs-diseases, etc.). This leaves us with two candidate methods: maximum entropy models (MEMMs) and conditional random fields (CRFs). Both are discriminative models, however MEMMs have label bias issues [15]: they proceed to a normalization at each state of the sequence whereas CRFs normalize the whole sequence. This is the reason why we opted for CRFs.

#### Experiments on the two models

We used the *F*_*tp*,*fp*_ metric [16], which definition is given in Equation 1, to assess the performance of the tested machine learning algorithms on both sequential and non-sequential representations towards the hospitalization prediction task.

Let *TN* be the number of negative instances correctly classified (True Negative), *FP* the number of negative instances incorrectly classified (False Positive), *FN* the number of positive instances incorrectly classified (False Negative) and *TP* the number of positive instances correctly classified (True Positive). Let *K* the number of folds used to cross-validate (in our experiment *K*=10), and _*f*_ the notation used to distinguish a fold related metric like the number of true positives from the sum of true positives across all folds.

$$TP_{f} = \sum\limits_{i=1}^{K}TP^{(i)} \quad FP_{f} = \sum\limits_{i=1}^{K}FP^{(i)} \quad $$

$$FN_{f} = \sum\limits_{i=1}^{K}FN^{(i)} $$

1 $$ F_{tp, fp}=\frac{2.TP_{f}}{2.TP_{f}+FP_{f}+FN_{f}}  $$

We rely on state of the art non-sequential algorithms available in the Scikit-Learn library [17] and in the CRF implementation of sklearn-crfsuite^2^. The optimized hyperparameters determined by nested cross-validation are as follows (hyperparameters search space is detailled between brackets, the continuous random variable was generated by scipy.stats.expon^3^):

- *SVC*, C-Support Vector Classifier, which implementation is based on libsvm [13]: The penalty parameter C ([continuous random variable]), the kernel used by the algorithm [linear, radial basis function kernel -RBF- or polykernel] and the kernel coefficient gamma [continuous random variable].
- *RF*, Random Forest classifier [12]: The number of trees in the forest [integer between 10 and 500], the maximum depth in the tree [integer between 5 and 30], the minimum number of samples required to split an internal node [integer between 1 and 30], the minimum number of samples required to be at a leaf node and the maximum number of leaf nodes [integer between 10 and 50].
- *LR*, Logistic Regression classifier [11]: The regularization coefficient C [continuous random variable] and the penalty used by the algorithm [l1 or l2].
- *CRFs*, Conditional Random Fields algorithm [18]: The regularization coefficients *c*1 and *c*2 [continuous random variable for both] used by the solver limited-memory BFGS (the default algorithm used in this library).

We evaluated our representations following the K-Fold method (with a *K* fixed at 10), a cross-validation strategy which allows us to test a classification algorithm across all the considered data. We optimized the hyperparameters of the machine learning algorithms used in this study with nested-cross validation [19] in order to avoid bias, and the exploration was done with random search [20]. The inner loop was executed with *L* fixed at 2 over 7 iterations, which corresponds to 14 fits by machine learning algorithms. This process ensures that the hyperparameters are optimized without introducing new biases, since the training, validation and testing sets are distinct at each step. This hyperparameter optimization step aims to improve the predictive power of the algorithms to better distinguish patients to be hospitalized from others. The different experiments were conducted on a Precision Tower 5810, 3.7GHz, 64GB RAM with a virtual environment under Python 3.5.4.

Table 3 presents the values of *F*_*tp*,*fp*_ obtained with the above described state of the art machine learning algorithms on the dataset *DS*_*B*_ shaped with our sequential and non sequential representations. The training time of CRFs with this model was expensive (22 hours with our protocol) and since it does not outperform logistic regression (best score with 0.85), we decided to consider only non-sequential EMR representation in our following experiments on the enrichment of vector representations with ontological knowledge.

**Table 3:** *F*_*tp*,*fp*_ of the selected classifiers on the balanced dataset *DS*_*B*_

*SVC*	*RF*	*LR*	*CRFs*
0.819	0.831	**0.850**	0.834

Best results entries have been highlighted in bold

### Predicting hospitalization from ontology-augmented representations of electronic medical records

Electronic medical records contain both structured data with fields relating to prescriptions and reasons for consultations, and also unstructured data such as free text. This section presents the different experiments we have conducted to perform a semantic enrichment of this data and the methods we designed to determine the relevant concepts in the assessment of hospitalization risk.

#### Ontology-augmented vector models of medical records

We reused the dataset *DS*_*B*_ to generate vectors as well as the non sequential text representations discussed in the previous section. Compared to the previous representation, here we proceed to the concatenation of the bag-of-words vector representations with a vector of concepts:

Let $V^{i}=\{w_{1}^{i},w_{2}^{i},..., w_{n}^{i}\}$ be the bag-of-words obtained from the textual data in the EMR of the *i*^*th*^ patient. Let $C^{i}=\{c_{1}^{i},c_{2}^{i},..., c_{m}^{i}\}$ be the bag-of-concepts (BOC) belonging to knowledge graphs and extracted from the EMR of the *i*^*th*^ patient. The data subject to extraction include both text fields listing drugs and pathologies with their related codes, and unstructured data from free texts such as observations. The vector representation of the *i*^*th*^ patient is the concatenation of *V*^*i*^ and *C*^*i*^: *x*^*i*^=*V*^*i*^⊕*C*^*i*^. More details about this representation can be found in [21]. The different machine learning algorithms that we tested to predict hospitalization from the enriched representation of EMRs will exploit these aggregated vectors. The resulting representations built are dense, most patients (instances) do not share the same features.

Concepts from knowledge graphs are considered as a token in a textual message. When a concept is identified in a patient’s medical record, this concept is added to the concept vector. This attribute will have as value the number of occurrences of this concept within the patient’s health record. For instance, the concepts ‘Organ Failure’ and ‘Medical emergencies’ (from DBpedia) are identified for ‘acute pancreatitis’, and the value for these attributes in our concept vector will be equal to 1.

**Fig. 5:**
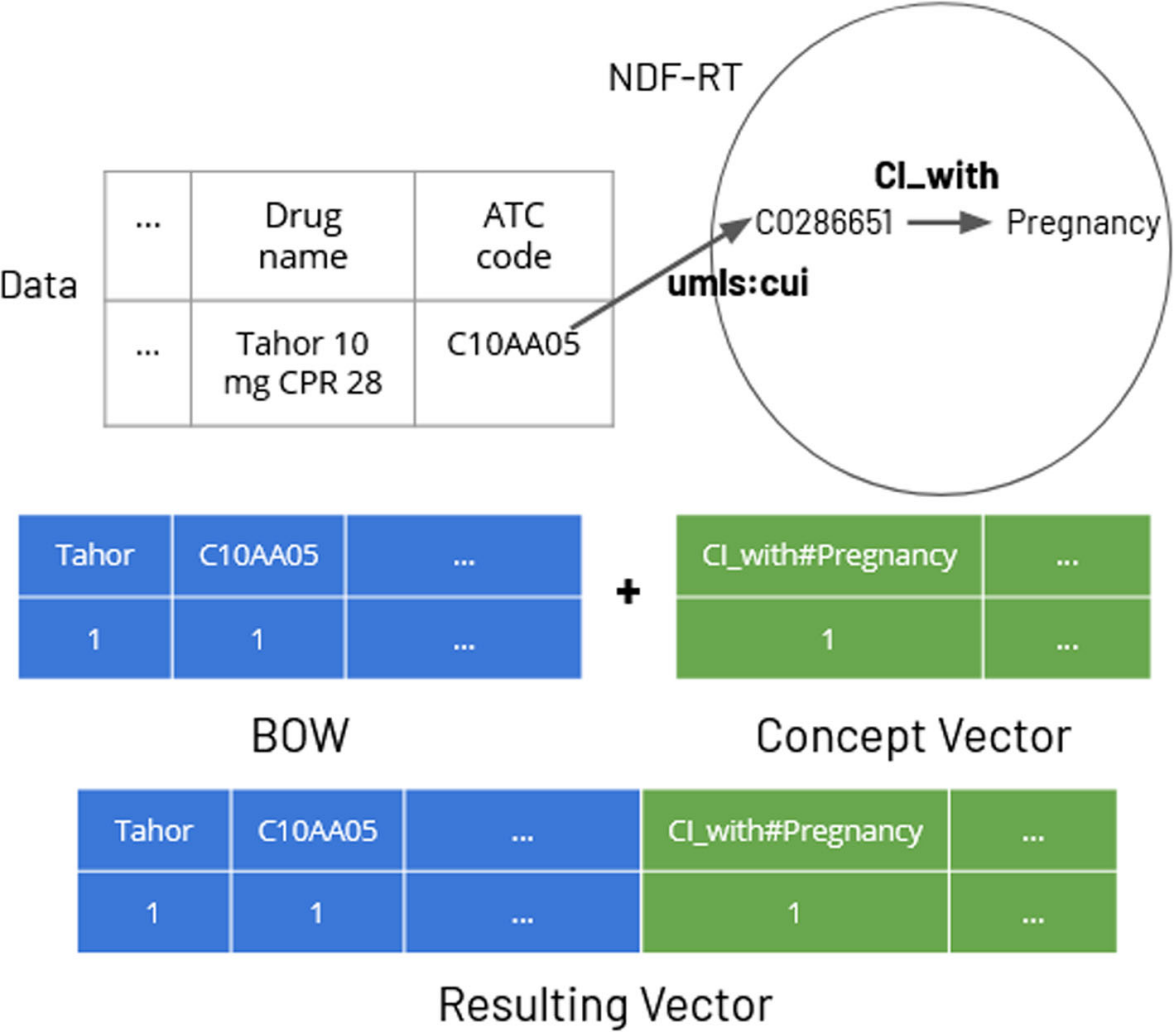
Concatenation of a bag-of-words representation *V* and a bag-of-concepts representation *C* of EMRs. In this example, we use the drug tahor whose main molecule is atorvastatin and we show how we extract and use one of these contraindicated effects (property CI_with) from the NDF-RT ontology

Similarly, if a property-concept pair is extracted from a knowledge graph (like in Wikidata and NDF-RT cases -features sets: +*wa*,+*wi*,+*wm* and +*d*-), it is added to the concept vector. For instance, in vectors exploiting NDF-RT (enrichment with +*d*), we find the couple consisting of CI_with as a property -contraindicated with- and the name of a pathology or condition, for instance ‘Pregnancy’ (triple found for the drug ‘Tahor’, main molecule ‘Atorvastatin’). The resulting feature of the BOC vector will be named after the property-concept pair. This example is depicted in Fig. 5 where we show how to concatenate the *V*^*i*^ and *C*^*i*^ vectors.

#### Extraction of relevant knowledge for prediction

**Fig. 6:**
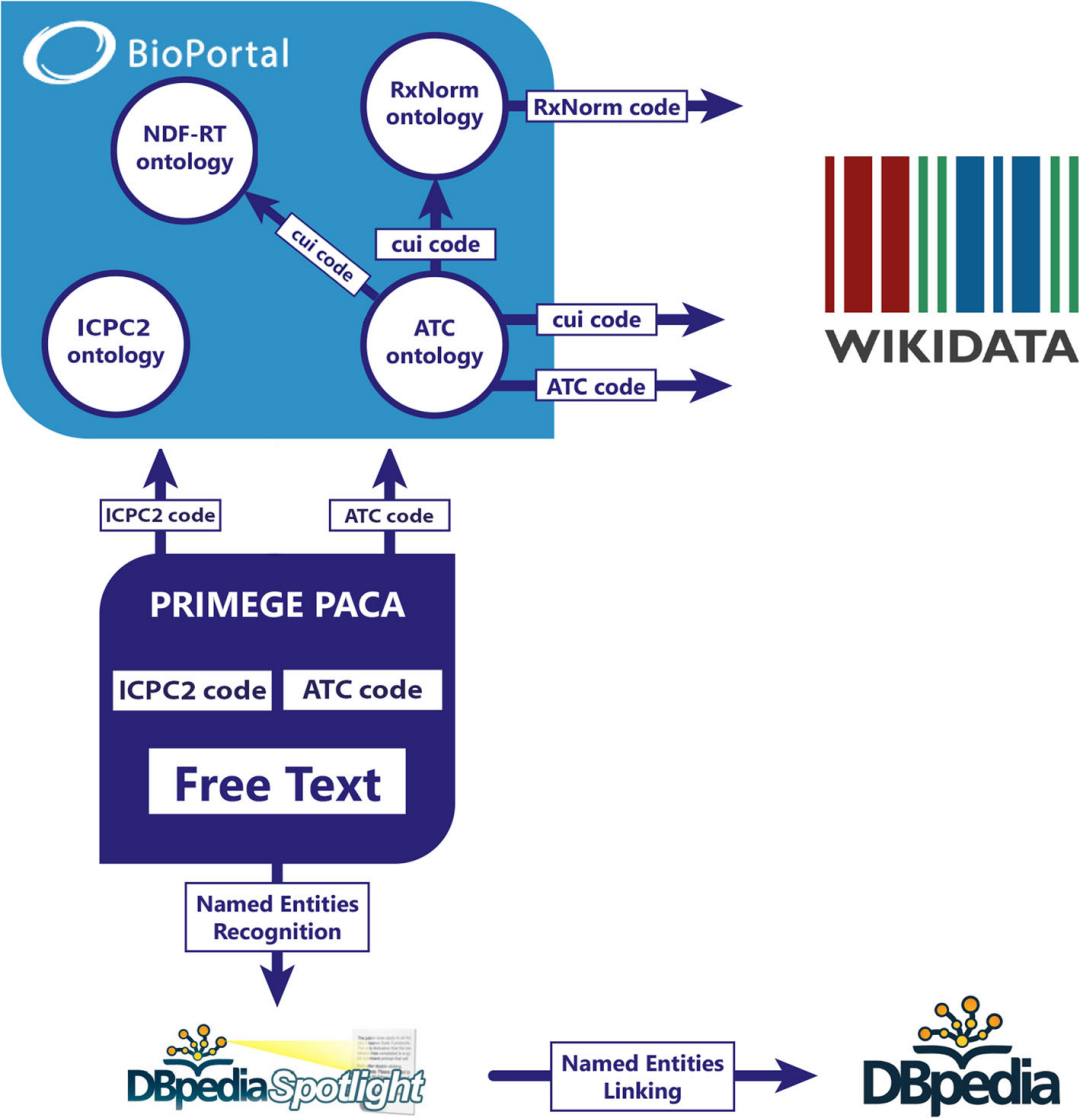
Workflow to link ATC codes, ICPC-2 codes and named entities in the EMRs with medical domain ontologies and with the knowledge graphs Wikidata and DBpedia

In this section, we detail how to extract knowledge from both structured and unstructured data in EMRs referring to both specialized and cross-domain knowledge graphs. The knowledge extracted will be used to build the BOC. The workflow is shown in Fig. 6.

*Knowledge extraction based on specialized ontologies.* We leveraged structured data to query OWL^4^ and SKOS^5^ representations of domain-specific ontologies and thesaurus. From the ICPC-2^6^ codes linked to reasons of consultations and the ATC^7^ codes used for the drugs prescribed to patients present in the PRIMEGE database we generate links to the corresponding resources in the ICPC-2 and ATC ontologies available through BioPortal. We also generate links to the NDF-RT^8^ ontology which contains specifications about drug interactions. The choice of these ontologies came naturally since the ATC and ICPC-2 codes are adopted in the PRIMEGE database, and NDF-RT contains additional information on drugs that capture interactions between drugs, diseases, mental and physical conditions.

For each ATC or ICPC-2 code present in a medical record, we extracted its super classes in its corresponding ontology, by using a SPARQL query with a rdfs:subClassOf property path. For instance, ‘tenitramine’ (ATC code: C01DA38) has as super class ‘Organic nitrates used in cardiac disease’ (ATC code: C01DA) which itself has as super class ‘VASODILATORS USED IN CARDIAC DISEASES’ (ATC code: C01D) which has for super class ‘CARDIAC THERAPY DRUGS’ (ATC code: C01). As for ICPC-2 code, the ontology does not have a high level of granularity, so it is only possible to extract one super class per diagnosed health problem or identified care procedure.

The link to NDF-RT resources was achieved via the CUI codes retrieved in the ATC ontology (with property umls:cui). The successor of NDF-RT is MED-RT^9^ (Medication Reference Terminology), but there is not yet a Semantic Web formalization.

*Knowledge extraction based on cross-domain knowledge graphs.DBpedia knowledge graph.* DBpedia^10^ is a crowdsourced extraction of knowledge pieces from Wikipedia articles^11^ and formalized with Semantic Web languages. DBpedia’s applications are varied and can range from organizing content on a website to uses in the domain of artificial intelligence.

We identified named entities in free-text fields of EMRs by using both a dictionary based approach to handle abbreviations and the semantic annotator DBpedia Spotlight [22]. We focused on the subject of the resources identified by DBpedia Spotlight (retrieved by querying DBpedia for the values of property dcterms:subject).

Initially, together with domain experts, we carried out a manual analysis of the named entities detected on a sample of approximately 40 consultations with complete information and selected 14 SKOS top concepts designating medical aspects relevant to the prediction of hospitalization, as they relate to severe pathologies. These concepts are listed in Table 4.

**Table 4:** List of manually selected concepts to determine a hospitalization. These concepts are translated from French to English (the translation does not necessarily exist for the English DBpedia chapter)

Speciality	Labels
Oncology	Neoplasm stubs, Oncology, Radiation therapy
Cardiovascular	Cardiovascular disease, Cardiac arrhythmia
Neuropathy	Neurovascular disease
Immunopathy	Malignant hemopathy, Autoimmune disease
Endocrinopathy	Medical condition related to obesity
Genopathy	Genetic diseases and disorders
Intervention	Surgical removal procedures, Organ failure
Emergencies	Medical emergencies, Cardiac emergencies

**Fig. 7:**
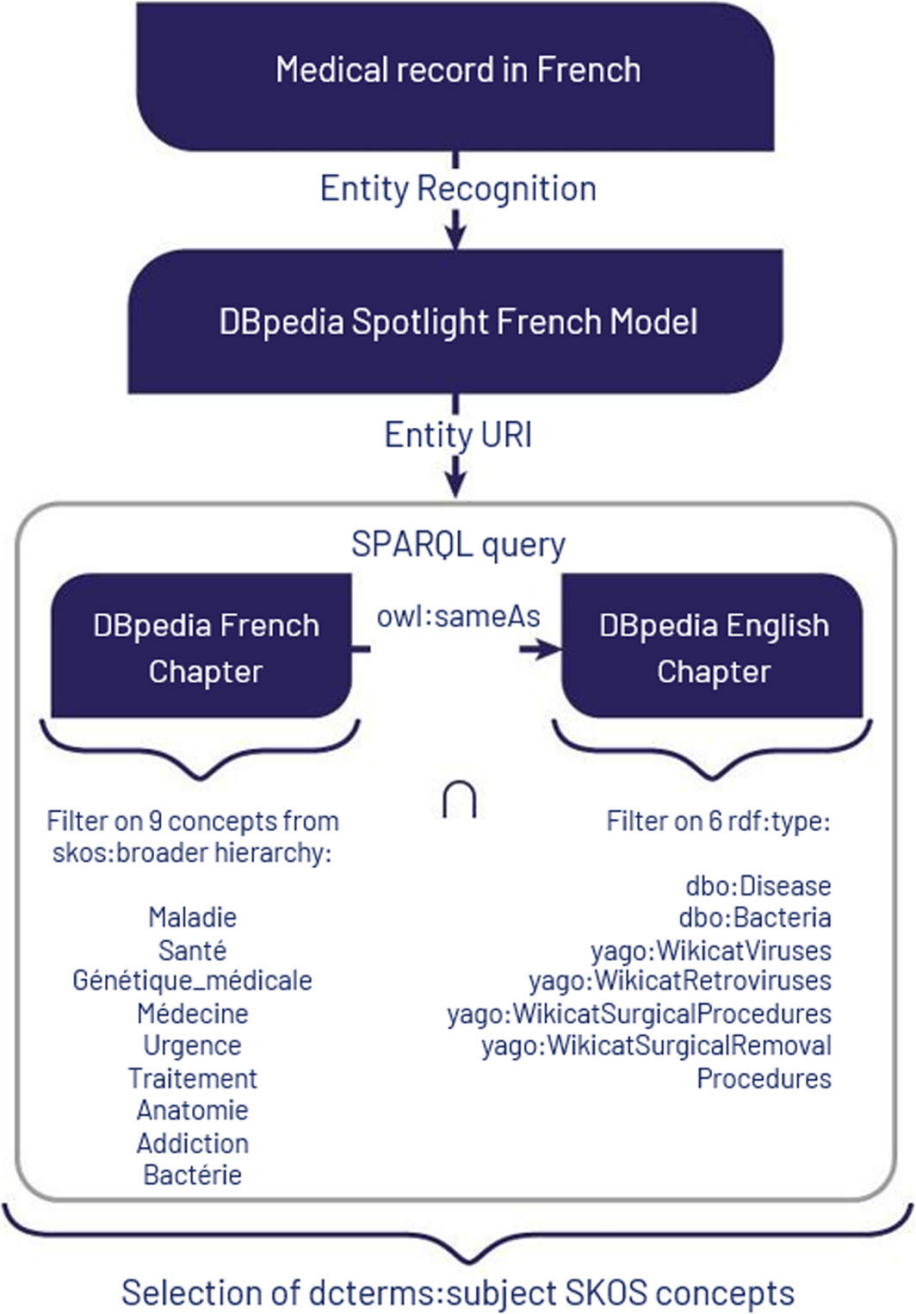
Workflow to extract candidate subjects from EMRs using DBpedia

We now propose an automated and more integrative approach to limit the scope of possible entities identified by DBpedia Spotlight and bind them to the medical field. To do so, we formalized and executed two constraints modeled by a federated SPARQL query shown in Listing 1. Figure 7 represents the workflow using this query.

The first SERVICE clause of the SPARQL query carried out on the French chapter of DBpedia retrieves entities identified by DBpedia Spotlight and belonging to the medical domain: they are the labels (property skos:prefLabel) of resources having as subject (property dcterms:subject^12^) a concept that belongs to the SKOS hierarchy (property skos:broader) of one of the French terms for disease, health, medical genetics, medicine, urgency, treatment, anatomy, addiction and bacteria.

The second SERVICE clause of the query further refines the set of retrieved entities by constraining them to be equivalent (property owl:sameAs) to English entities belonging to at least one of the following medical classes (property rdf:type): dbo:Disease, dbo:Bacteria, yago:WikicatViruses, yago:WikicatRetroviruses, yago:WikicatSurgicalProcedures, yago:WikicatSurgicalRemovalProcedures. We empirically restricted to these few classes and discarded many other medical classes that would introduce noise. For instance dbo:Drug, dbo:ChemicalCoumpound, dbo:ChemicalSubstance, dbo:Protein, or yago:WikicatMedicalTreatments allow to retrieve entities related to chemical compounds, thus entities that can range from drugs to plants or fruits. Types referring to other living things such as umbel-rc:BiologicalLivingObject, dbo:Species or dbo:AnatomicalStructure would select entities describing a wide range of species since the scope of these types is not restricted to humans, and includes bacteria, viruses, fungus or parasites affecting humans. Likewise, the class dbo:AnatomicalStructure was used for describing different things in the previous versions of DBpedia (i.e., ‘Barrier layer (oceanography)’, ‘Baseball doughnut’, etc.). We also discarded biomedical types in the yago namespace defined in DBpedia^13^ which URI ends by an integer (e.g., http://dbpedia.org/class/yago/Retrovirus101336282) because they are too numerous and too semantically close to each other.

In the end, the entities retrieved by this SPARQL query on DBpedia are used to build the vector representation of EMRs from the features extracted from their text fields.


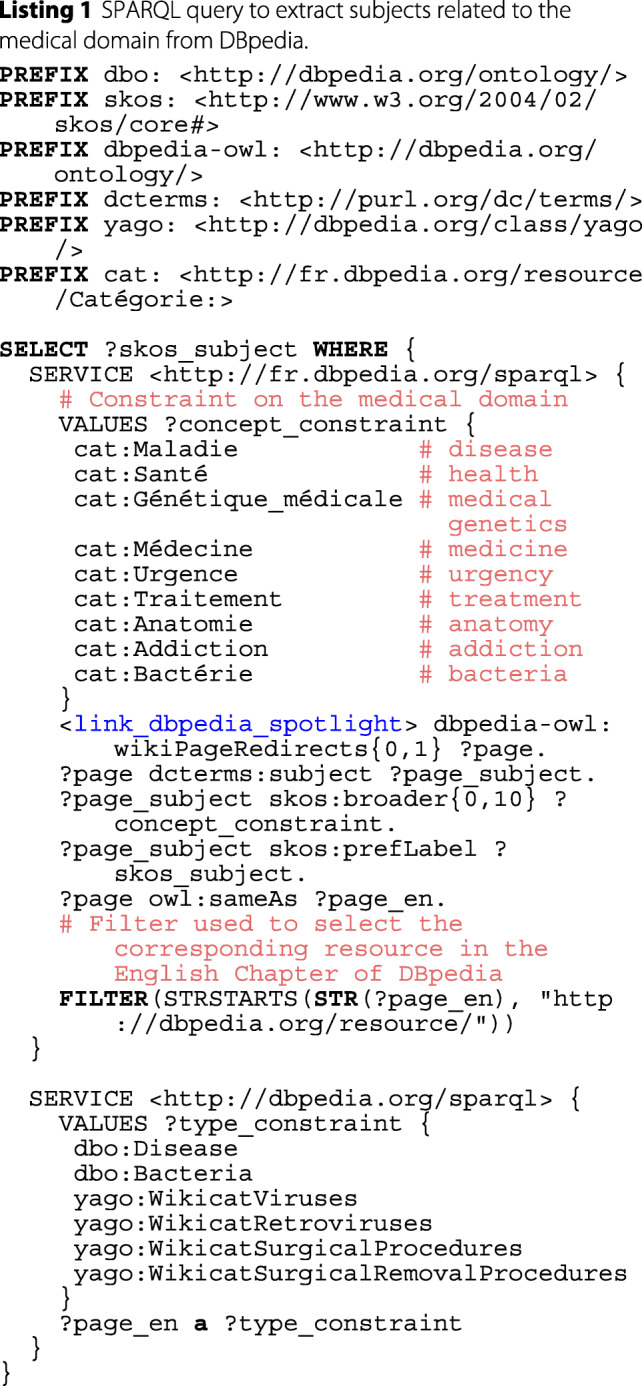


Table 5 presents two examples of observations with their extracted DBpedia concepts. In the first one, the expression ‘insuffisance cardiaque’ (heart failure) leads to the entity dbpedia-fr:insuffisance_cardiaque^14^ (cardiac insufficiency) which has for dcterms:subject category-fr:Défaillance_d’organe^15^ (organ failure) and category-fr:Maladie_cardiovasculaire (cardiovascular disease). In the second observation, the expression ‘kyste’ (cyst) leads to the entity dbpedia-fr:Kyste_(médecine) which has for dcterms:subject category-fr:Anatomo-pathologie_des_tumeurs (neoplasm stubs).

**Table 5:** Examples of concepts extracted from free text in EMRs with our approach using a dictionary to handle abbreviations (brackets indicate corrections including typos and abbreviations), using DBpedia Spotlight to recognize entities, and querying DBpedia to retrieve relevant medical concepts

	Patient 1	Patient 2
French	prédom à gche - insuf vnse ou - pas signe de phlébite - - ne veut pas mettre de bas de contention et ne veut pas aumenter le lasilix... -	procédure FIV - - transfert embryon samedi dernière - a fait hyperstimulation ovarienne; rupture de - - asthénie, - - dleur abdo, doulleur à la palpation ++ - - voit gynéco la semaine prochaine pr controle betahcg, echo-
English (Translation)	predom[inates] on the l[e]ft, venous or , no evidence of phlebitis, does not want to wear compression stockings and does not want to increase the lasix	In vitro fertilization procedure, embryo transfer last Saturday, did ovarian hyperstimulation, rupture, asthenia abdominal [pain], [pain] on palpation ++, will see a gyneco[logist] next week [for] a beta HCG, echo check-up
Concepts	Cardiovascular disease, Organ failure	Neoplasm stubs

*Wikidata knowledge graph.* Wikidata^16^ is an open knowledge base, collaboratively edited, that centralizes data from projects of the Wikimedia Foundation^17^. For specific datasets in the biomedical domain, Wikidata also benefits from automatic laboratory submissions of the latest research works. For Wikidata, we focused on augmenting our data with information extracted from the properties linked to drugs as we did with the NDF-RT and ATC ontologies. To link to Wikidata, we used the ATC (property wdt:P267), CUI UMLS (property wdt:P2892) and CUI RxNorm codes (property wdt:P3345), since Wikidata contains at least one of them for each drug. To use RxNorm, we proceed in a similar way as for NDF-RT with the CUI codes contained in the ATC ontology. Thus, we queried the SPARQL endpoint of Wikidata^18^ to extract knowledge related to drugs, by using three properties: ‘subject has role’ (property wdt:P2868), ‘significant drug interaction’ (property wdt:P2175), and ‘medical condition treated’ (property wdt:P769).

*Inter-rater reliability of concept annotation.* Now that we have shown how we extracted knowledge from knowledge graphs, we investigate the particular case of the relevance of DBpedia concepts in predicting hospitalization. We aim to distinguish knowledge that introduces noise from knowledge beneficial for the prediction and establish a strategy to improve decision making.

285 concepts from DBpedia were extracted from the query in Listing 1 and were independently annotated by two general practitioners and one biologist. The different annotations were compared with the Krippendorff’s *alpha* metric [23]. We also used the correlation metric^19^ to compare pairs of vectors from human or machine annotation.

The initial Krippendorff’s *α* score between the three annotators is 0.51, and the score between the two GPs is 0.27. Some expressions were problematic because they are compound (composed terms) creating terminological conflict by including one or several other terms. As a result they were annotated in the same way by an annotator. It was for instance the case for compounds starting with ‘Biology’ (i.e., ‘Biology in nephrology’, ‘Biology in hematology’, etc.), ‘Screening and diagnosis’ (i.e., ‘Infectious disease screening and diagnosis’, ‘Screening and diagnosis in urology’, etc.), ‘Pathophysiology’ (i.e., ‘Pathophysiology of the cardiovascular system’, ‘Pathophysiology in hematology’, etc.), ‘Psychopathology’ (i.e., ‘Psychoanalytical psychopathology’, ‘Psychopathology’), ‘Clinical sign’ (i.e., ‘Clinical signs in neurology’, ‘Clinical signs in otorhinolaryngology’, etc.), ‘Symptom’ (i.e., ‘Symptoms in gynecology’, ‘Symptom of the digestive system’, etc.) and ‘Syndrome’ (i.e., ‘Syndrome in endocrinology’, ‘Syndrome in psychology or psychiatry’, etc.). Even by excluding these compounds from the considered concepts, which brings us back to 243 concepts, the three annotators obtained a Krippendorff’s *α* score of 0.66, and 0.52 for the inter-rater reliability between the two GPs.

From the 285 concepts, on average 198 were estimated as relevant to the study of patients’ hospitalization risks by experts: the two GPs estimated respectively 217 and 181 concepts as relevant, and the biologist 196 concepts.

Artstein and Poesio [24] states that such a score is insufficient to draw conclusions. This shows to what extent this annotation task is more difficult than it may seem, in particular because identifying the entities involved in the hospitalization of a patient is subjective and it is therefore hard to find an agreement.

Automatically selecting these concepts can be a way to find a consensus based on data. This is the reason why in the following sections, we generated vectors where knowledge was selected by machine annotations through feature selection and we compare them to the results of human annotations.

#### Experiments

*Experimental protocol.* Vector representations were evaluated by nested cross-validation [19], with an external loop with a *K* fixed at 10 and for the internal loop a *L* fixed at 3. The exploration of hyperparameters was performed with random search [20] with 150 iterations. The HP EliteBook was used to generate vector representations and to deploy DBpedia Spotlight as well as domain-specific ontologies with the Corese Semantic Web Factory^20^ [25].

The different experiments were conducted on a HP EliteBook 840 G2, 2.6 hHz, 16 GB RAM with a virtual environment under Python 3.6.3 as well as a Precision Tower 5810, 3.7GHz, 64GB RAM with a virtual environment under Python 3.5.4. Like in the experiment reported in the previous section, we rely on the algorithms available in the Scikit-Learn library, with *SVC*, *RF*, *LR* and we optimized the same hyperparameters.

We used the *F*_*tp*,*fp*_ metric [16], defined in Equation 1, to assess the performance of selected machine learning algorithms using our vector representations of EMRs enriched with ontological knowledge. We also computed *PR*_*avg*_,*RE*_*avg*_,*F*1_*avg*_,*AUC*_*avg*_ and their standard error variations for *LR*, the algorithm that performs best.

Since our experimental protocol uses cross-validation, the training sets overlap, which violates the independence assumption in many statistical tests in the literature [26]. Thus, we opted for the correction of dependent Student’s t test [27] that addresses this issue to confirm the statistical impact of the features extracted from knowledge graphs. It is defined as follows:

$$t=\frac{\frac{1}{n}\sum_{j=1}^{n}x_{j}}{\sqrt{\left(\frac{1}{n}+\frac{n_{2}}{n_{1}}\right)\widehat{\sigma}^{2}}} $$

where *x*_*j*_=*A*_*j*_−*B*_*j*_, with *A*_*j*_ the metric obtained at the *j*^*th*^ fold in the set of metrics *A* and *B*_*j*_ an another metric in *B*, *A* and *B* are the vectors of size *n* produced by the two compared methods. Thus *x*_*j*_ represents the difference between two evaluations in the fold *j* (here we used the metrics obtained with the baseline against the metrics of other features sets), *n*_2_ is the number of testing folds (in our case *n*_2_=1), *n*_1_ is the number of training folds (in our case *n*_1_=9) and $\widehat {\sigma }^{2}$ is the sample standard deviation on *x*.

*Feature sets variations and notation.* We aimed to measure the impact of enriching the vector representations of EMRs with different features extracted from knowledge graphs when predicting hospitalization. We detail below the notations used to refer to the different vector representation evaluated in our experiments:

- *baseline*: bag-of-words representation of EMRs, no ontological enrichment is made on EMR data.
- +*t* : refers to an enrichment with concepts from the OWL-SKOS representation of ICPC-2.
- +*c*: refers to an enrichment with concepts from the OWL-SKOS representation of ATC, the number or number interval indicates the different hierarchical depth levels used.
- +*wa*: refers to an enrichment with Wikidata’s ‘subject has role’ property (wdt:P2868).
- +*wi*: refers to an enrichment with Wikidata’s ‘significant drug interaction’ property (wdt:P769).
- +*wm*: refers to an enrichment with Wikidata’s ‘medical condition treated’ property (wdt:P2175).
- +*d*: refers to an enrichment with concepts from the NDF-RT OWL representation, _prevent_ indicates the use of the may_prevent property, _treat_ the may_treat property and _CI_ the CI_with property.

Here, we detail the additional notations to refer to vector representations built from the different methods of selection of concepts from DBpedia. For features sets other than +*s*∗ and +*s*, we evaluated the impact of the selection of concepts extracted from DBpedia, whether this feature selection process is performed by machines or humans. This is to observe whether various feature selection methods are relevant to improve the prediction of hospitalization and thus have an impact on reducing the noise that knowledge graphs can bring:

- The +*s*∗ notation refers to an approach using the enrichment of representations with concepts among the list of the 14 manually selected concepts (see Table 4) from DBpedia. This approach does not exploit all text fields to extract knowledge from DBpedia, these fields are related to the patient’s own record with: the patient’s personal history, allergies, environmental factors, current health problems, reasons for consultations, diagnosis, drugs, care procedures, reasons for prescribing drugs and physician observations.
- The +*s* notation refers to an approach using the enrichment of representations with concepts among the list of the 14 manually selected concepts (see Table 4) from DBpedia. This approach uses all text fields to identify entities with: the patient’s personal history, family history, allergies, environmental factors, past health problems, current health problems, reasons for consultations, diagnosis, drugs, care procedures, reasons for prescribing drugs, physician observations, symptoms and diagnosis.
- +*s*∗*T* refers to an enrichment with the labels of concepts automatically extracted from DBpedia with the help of the SPARQL query in Listing 1, 285 concepts are thus considered with this approach. Like all representations starting with prefix +*s*∗, concepts were extracted from fields related to the patient’s own record: history, allergies, environmental factors, current health problems, reasons for consultations, diagnosis, drugs, care procedures, reasons for prescribing drugs and physician observations.
- +*s*∗∩ refers to an enrichment with a subset of the labels of concepts automatically extracted from DBpedia acknowledged as relevant by *at least one* expert human annotator. This approach uses the same text fields as the previous features set.
- +*s*∗∪ refers to an enrichment with a subset of the labels of concepts automatically extracted from DBpedia acknowledged as relevant by **all** the expert human annotators. This approach uses the same text fields as the previous features sets.
- +*s*∗*m* refers to an enrichment with a subset of the labels of concepts automatically selected by using a feature selection algorithm. We chose the Lasso algorithm [28] and we executed it *within* the internal loop of the nested cross-validation (with L, the number of folds fixed at 3) in the global machine learning algorithm chosen to predict hospitalization. This approach uses the same text fields as the previous features sets.
- +*sm* uses the same enrichment procedure of +*s*∗*m* to automatically select a subset of the labels of concepts. Contrary to the other features sets built with DBpedia, this one uses all text fields, so in addition to the ones from *s*∗, we consider: family history, past health problems, symptoms.
- +*sm*∩ uses a subset of +*sm* with concepts selected by feature selection in **all** the 10 folds (external loop). This approach uses the same text fields as the previous features set. In total, it considers 14 different concepts (or 19 concepts if we consider that 2 concepts with the same name but different prefixes are different).
- +*sm*∪ uses a subset of +*sm* with concepts selected by feature selection in **at least one** fold out of 10 (external loop). This approach uses the same text fields as the previous features sets. In total, it considers 51 different concepts (or 63 concepts when taking into account prefixes).

*Results.* First, we compared human and machine annotations with the generalization of the vectors (*U*_1_ or +*sm*∪ approach) produced through machine annotations, since the concepts selected with feature selection and nested cross validation may differ from one training set to another. Table 6 displays correlation metric values between experts and machine annotators (its value ranges from 0 to 2, meaning that 0 is a perfect correlation, 1 no correlation and 2 perfect negative correlation). We compare pairs of vectors in this table, if they are deemed relevant, irrelevant or not annotated (in the case of human annotation) to study the patient’s hospitalization risks.

**Table 6:**
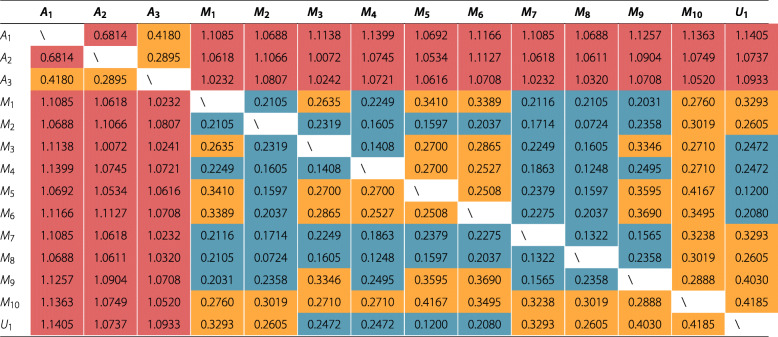
Correlation metric ($1-\frac {(u-\bar {u}).(v-\bar {v})}{{\vert \vert u-\bar {u}\vert \vert }_{2}{\vert \vert v-\bar {v}\vert \vert }_{2}}$, with $\bar {u}$, the mean of elements of *u*, and respectively $\bar {v}$, the mean of elements of *v*) computed on the 285 concepts. *A*_1_ to *A*_3_ refers to human annotators and *M*_1_ to *M*_10_ refers to machine annotators through feature selection annotation on the +*sm* approach (considering the 10 K-Fold). *U*_1_ (or +*sm*∪) is the union of subjects from the sets *M*_1_ to *M*_10_. Cells in  are strictly superior to 0.5, cells in  are between 0.25 and 0.5, cells in  are strictly inferior to 0.25

Then, Table 7 reports the results for each representation we tested on the *DS*_*B*_ dataset with the *F*_*tp*,*fp*_ metric. Table 8 shows the average metrics we computed and their standard deviation errors to give more details on the behavior of the enriched vectors on the best performing machine learning algorithm, the logistic regression.

**Table 7:** *F*_*tp*,*fp*_ for the different vector sets considered on the balanced dataset *DS*_*B*_ under logistic regression

Features set	*SVC*	*RF*	*LR*	Average
*baseline*	0.8270	**0.8533**	0.8491	0.8431
+*t*	0.8239	0.8522	**0.8545**	0.8435
+*c*_1_	0.8235	0.8433	0.8453	0.8245
+*c*_1−2_	0.8254	0.8480	0.8510	0.8415
+*c*_2_	0.8348	0.8522	0.8505	**0.8458**
+*d*_*prevent*_	0.8254	0.8506	0.8479	0.8413
+*d*_*treat*_	0.8338	0.8472	0.8481	0.8430
+*d*_*CI*_	0.8281	0.8498	0.8460	0.8413
+*wa*	0.8223	0.8468	**0.8545**	0.8412
+*wi*	0.8149	0.8484	0.8501	0.8378
+*wm*	0.8221	0.8453	0.8458	0.8377
+*s*	0.8221	0.8522	0.8485	0.8409
+*s*∗	0.8339	0.8449	0.8514	0.8434
+*s*∗*T*	0.8214	0.8492	0.8388	0.8365
+*s*∗∩	0.8262	0.8521	0.8432	0.8405
+*s*∗∪	0.8270	0.8467	0.8445	0.8394
+*s*∗*m*	0.8363	0.8547	**0.8642**	0.8517
+*sm*	0.8384	0.8541	**0.8689**	0.8538
+*sm*∩	NA	NA	**0.8662**	NA
+*sm*∪	NA	NA	**0.8714**	NA

Best results entries have been highlighted in bold

**Table 8:** *PR*_*avg*_,*RE*_*avg*_,*F*1_*avg*_,*AUC*_*avg*_ and their standard error variations computed between each folds for the different vector sets considered on the balanced dataset *DS*_*B*_ under logisitc regression

Features set	*PR*_*avg*_	*RE*_*avg*_	*F*1_*avg*_	*AUC*_*avg*_	*STD*(*PR*)	*STD*(*RE*)	*STD*(*F*1)	*STD*(*AUC*)
*baseline*	0.8786	0.8236	0.8490	0.8551	0.0473	0.0484	0.0353	0.0334
+*t*	0.8819	0.8306	0.8546	0.8600	0.0439	0.0344	0.0283	0.0280
+*c*_1_	0.8798	0.8152	0.8453	0.8523	0.0435	0.0432	0.0323	0.0309
+*c*_1−2_	0.8775	0.8278	0.8511	0.8565	0.0442	0.0400	0.0320	0.0311
+*c*_2_	0.8795	0.8250	0.8508	0.8565	0.0442	0.0335	0.0315	0.0309
+*d*_*prevent*_	0.8756	0.8235	0.8478	0.8537	0.0420	0.0457	0.0322	0.0302
+*d*_*treat*_	0.8740	0.8251	0.8482	0.8538	0.0403	0.0353	0.0321	0.0309
+*d*_*CI*_	0.8721	0.8236	0.8462	0.8517	0.0506	0.0449	0.0375	0.0366
+*wa*	0.8816	0.8306	0.8545	0.8600	0.0451	0.0443	0.0348	0.0334
+*wi*	0.8766	0.8264	0.8498	0.8559	0.0377	0.0506	0.0348	0.0321
+*wm*	0.8730	0.8221	0.8458	0.8516	0.0436	0.0430	0.0320	0.0311
+*s*	0.8766	0.8235	0.8484	0.8544	0.0442	0.0457	0.0357	0.0337
+*s*∗	0.8799	0.8264	0.8502	0.8572	0.0446	0.0446	0.0341	0.0325
+*s*∗*T*	0.8755	0.8025	0.8375	0.8466	0.0256	0.0634	0.0405	0.0329
+*s*∗∩	0.8800	0.8094	0.8420	0.8507	0.0269	0.0597	0.0368	0.0322
+*s*∗∪	0.8734	0.8177	0.8433	0.8508	0.0282	0.0633	0.0399	0.0337
+*s*∗*m*	0.8929	0.8376	0.8639	0.8642	0.0259	0.0398	0.0280	0.0258
+*sm*	0.9001	0.8404	0.8686	0.8744	0.0267	0.0431	0.0287	0.0261
+*sm*∩	0.8966	0.8389	0.8660	0.8717	0.0349	0.0427	0.0296	0.0277
+*sm*∪	0.9008	0.8445	0.8712	0.8765	0.0283	0.0378	0.0257	0.0240

**Fig. 8:**
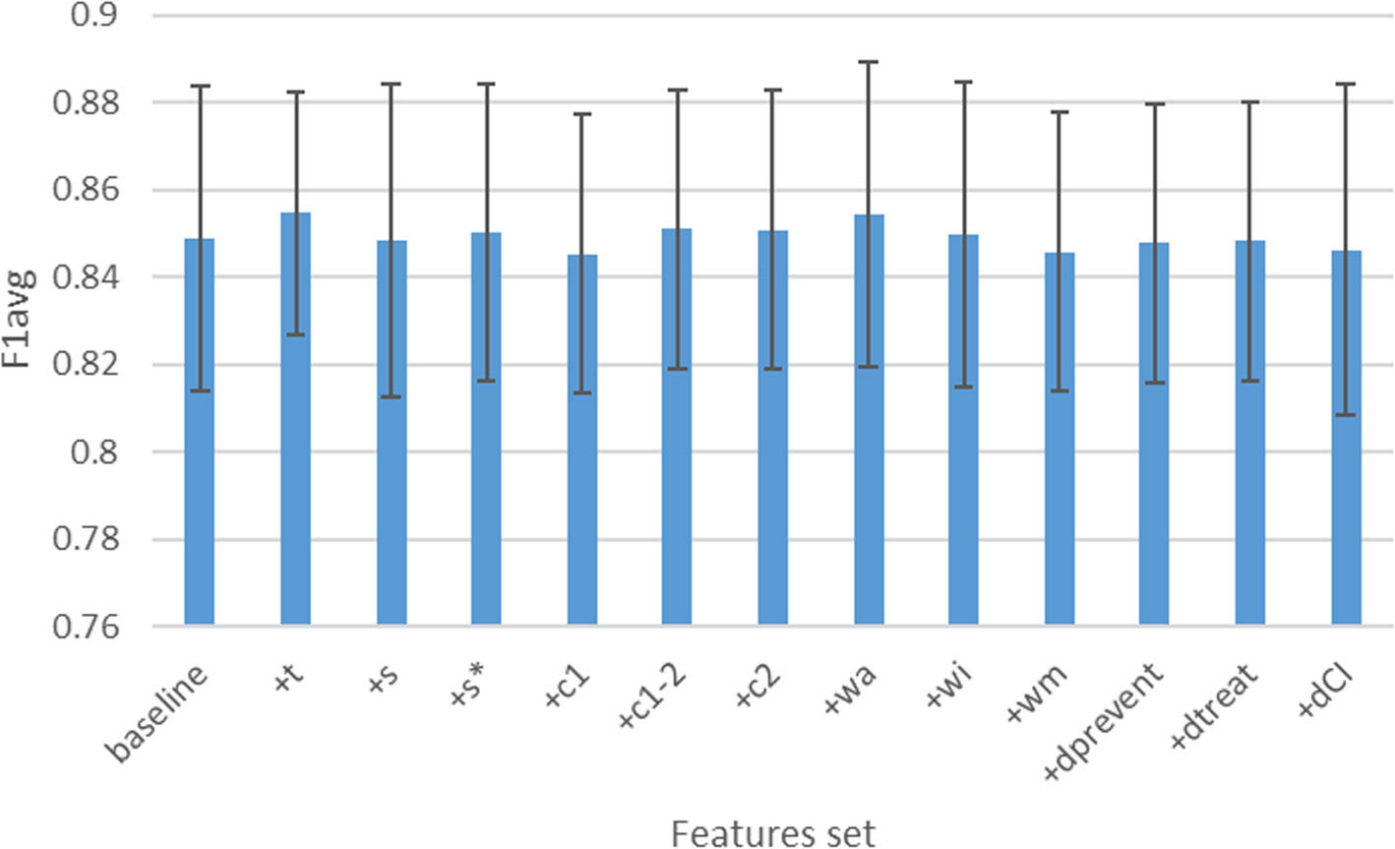
Histograms that represent the average F1 score (y-axis) and standard deviations under logistic regression for most of the vector sets considered in Table 7 (x-axis)

Figure 8 shows the average F1 score (average between the different F1 scores obtained by cross-validation) and standard deviations associated to the vector sets under logistic regression considered in Table 7. By comparing this figure with the above-mentioned table, it appears that, contrary to the trend shown in the table, there is no approach that performs better than another. Overall, in 6 to 8 out of 10 folds for SVMs a linear kernel was chosen, and in 2 to 4 out of 10 folds an RBF kernel was selected.

**Fig. 9:**
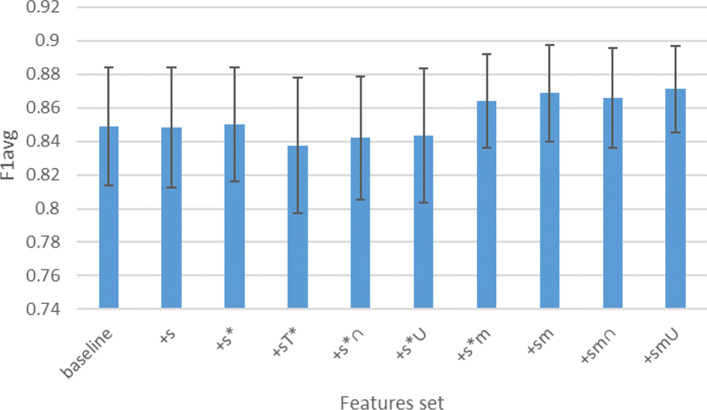
Histograms that represent the average F1 score (y-axis) and standard deviations under logistic regression for the vector sets considered in Table 7 (x-axis)

Figure 9 shows the average F1 scores and standard deviations under logistic regression associated to the vector sets derived from DBpedia considered in Table 7. Compared to other approaches, a slight improvement in the results is noticeable with automated feature selection approaches.

Table 9 shows the t-value/*p*-value pairs obtained with the F1 metric on each observation on different vector sets compared to the baseline. The corrected Student’s t test rejects the null hypothesis on the +*sm*∪ approach (with a t-value of 2.23 and a *p*-value of 0.05), the approach that consists in considering the union of concepts of +*sm* and which relies on DBpedia. This approach also obtained the best *F*_*tp*,*fp*_ (0.8714).

**Table 9:** t-value/*p*-value pairs on F1 for different vector sets considered on the balanced dataset *DS*_*B*_

Features set	t-value/*p*-value (on F1)
+*wa*	-1.06/0.32
+*sm*	-1.57/0.151
+*sm*∩	-1.62/0.139
+*sm*∪	**-2.23/0.05**

Best results entries have been highlighted in bold

*Discussion.* In terms of feature selection, we observe in Table 6 wide variations between human annotators and machine annotators (maximum of 1.1399 between *A*_1_ and *M*_4_), whereas between annotators of a specific group this margin is not as significant (maximum of 0.6814 for humans and maximum of 0.4185 for machines). The union of concepts *U*_1_ (or +*sm*∪) selected by machine annotators is really similar to *M*_5_, since they have score of 0.12.

Among the 51 concepts selected with +*sm*∪ (63 if we consider the provenance prefix), generic knowledge was selected such as ‘Medical Terminology’, one possibility could be that the general practitioner uses a technical terminology in a situation involving a complex medical case. Numerous concepts related to patient’s mental state appear to be a cause of hospitalization. Different concepts related to the allergy and infectious diseases were selected. Concepts related to the cardiovascular system are widely represented within this set. The only concept retrieved in the family history of the patient, with the exception of ‘Medical Terminology’ is ‘Diabetes’. Among the concepts automatically selected through feature selection, there are concepts initially considered irrelevant by the human experts (e.g., the concept ‘Medical Terminology’), they were finally reviewed as relevant in light of the explanation provided by the machine learning algorithm. These explanations are summarized in Table 10 with the corresponding concepts in English.

**Table 10:** Concepts involved in the hospitalization prediction among the 51 selected concepts of +*sm*∪

Source	Concept	Concept (Translated)
Generic knowledge	Terme médical	Medical terminology
Patient’s mental state	Antidépresseur, Dépression (psychiatrie), Psychopathologie, Sémiologie psychiatrique, Trouble de l’humeur	Antidepressant, Major depressive disorder, Psychopathology, Psychiatric assessment, Mood disorder
Infectious disease	Infection ORL, Infection urinaire, Infection virale, Virologie médicale	ENT infection, Urinary tract infection, Viral Infection, Clinical virology
Cardiovascular system	Dépistage et diagnostic du système cardio-vasculaire, Maladie cardio-vasculaire, Physiologie du système cardio-vasculaire, Signe clinique du système-cardiovasculaire, Trouble du rythme cardiaque	Screening and diagnosis of the cardiovascular system, Cardiovascular disease, Physiology of the cardiovascular system, Clinical sign of the cardiovascular system, Cardiac arrhythmia
Family history	Diabète, Terme médical	Diabetes, Medical terminology

In terms of prediction, the difference between the different settings measured with *F*_*tp*,*fp*_ is quite small but noticeable, however the standard deviation is quite large between the different F1-score obtained between each fold for each features set (ranging from 0.03 to 0.04, knowing that the *baseline* is at 0.035) limiting the conclusions that can be drawn from these results.

We display most of the results with +*s*∗ concepts, those extracted from the patient’s owns records since the experiments show that the use of all the text fields introduce noises and therefore lesser results. However, feature selection according to the origin of the concept allows to select only the relevant concepts and so to improve the predictions. A feature selection step can thus improve the prediction of hospitalization by adding knowledge indirectly related to the patient’s condition while avoiding the introduction of noise, such as family history (approach +*sm*).

## Results

We summarize here the results of our experiments that were detailed over several other sections.

With vector representations solely based on text features (with both structured and unstructured text data) our experimentation on the prediction of hospitalization with conditional random fields did not outperform the results obtained with logistic regression, this is the reason why we rely on a non-sequential EMR representation in the evaluation of the enrichment with ontological knowledge (see Section “Predicting hospitalization from text-based representations of electronic medical records” / “Experiments on the two models” for more details).

Human annotators are less likely to reach a consensus than machine annotators using feature selection process in determining the most relevant features. Injecting ontological knowledge seems to improve in most cases hospitalization prediction. However, when considering the standard deviation, it is difficult to draw conclusions for some of the configurations as shown in Table 8.

The specific configuration +*sm*∪ selected by feature selection across all folds shows statistically significant improvement, indicating that in that case injecting ontological knowledge improves the results, provided that noisy features are discriminated (see Section “Predicting hospitalization from ontology-augmented representations of electronic medical records” / “Experiments” / “Results” for more details).

## Discussion

The results show that using features extracted from multiple knowledge graphs to enrich the vector representation of EMRs, together with a selection mechanism for that knowledge can further improve the prediction of patient hospitalization. In addition, we showed that the knowledge relevant to a task as specific as predicting hospitalizations is not limited to specialized ontologies; cross-domain graphs contain knowledge that can help machine learning algorithms in such a prediction task.

Coupling knowledge graphs with the medical records also represents an opportunity to provide more explanation of the algorithm’s decision. As a result the decision can also be based on that knowledge enriching the patient’s medical record.

## Conclusion

In this paper, we presented a method to combine knowledge from specialized or cross-domain knowledge graphs and text from EMRs. We also show how to be selective and not introduce noise in this input to predict hospitalization. We generated different vector representations of EMRs combining both concept vectors and bag-of-words representations using named entity recognition and we compared the predictive power of these representations with different machine learning algorithms.

As future work, a representation combing in a more structured way the text from the record and the knowledge graphs could be interesting, because the bags of words have the downside of losing semantics relations between the features. Additional medical knowledge may also be extracted by integrating other semantic annotators on Wikidata (such as entity-fishing^21^) and on domain specific knowledge graphs (such as General Architecture for Text Engineering -GATE-22 [29]) that can be deployed locally so as not to compromise the confidential nature of this data. Although we have investigated the issues raised by having free texts as input with DBpedia, many problems still need to be addressed including a better management of abbreviations and spelling mistakes, negation as well as the context in which a medical expression is used, exploiting the class hierarchy of concepts for the value of the dcterms:subject property. This also implies improving the recognition of different medical expressions (expressions composed of multiple medical terms, unrecognized cases due to the plural or feminine in these complex expressions, etc.). A potential direction would be to evaluate the impact of a feature selection step coupled with a combination of features extracted from several knowledge graphs.

Longer-term perspectives would be to work on the integration of more heterogeneous data such as biological values, to propose personalized medicine by suggesting the best treatments for a patient. Other possible directions of this work are to address specific pathologies (cardiovascular diseases, mental illness, etc.) and to estimate the risks related to pandemics.

## Appendix

+*sm*∪ with the logistic regression algorithm (*LR*) uses the following parameters:

- Fold 1: ’C’: 0.056049240151690681, ’penalty’: ’l2’.
- Fold 2: ’C’: 0.83617364781543058, ’penalty’: ’l2’.
- Fold 3: ’C’: 0.078134513655501683, ’penalty’: ’l2’.
- Fold 4: ’C’: 0.070037689307546724, ’penalty’: ’l2’.
- Fold 5: ’C’: 0.030094071461144355, ’penalty’: ’l2’.
- Fold 6: ’C’: 0.19901721018094651, ’penalty’: ’l2’.
- Fold 7: ’C’: 0.16012788113832127, ’penalty’: ’l2’.
- Fold 8: ’C’: 0.067362109991791305, ’penalty’: ’l2’.
- Fold 9: ’C’: 0.034161307706627134, ’penalty’: ’l2’.
- Fold 10: ’C’: 0.055643396004174048, ’penalty’: ’l2’.

+*sm* with the c-support vector classifier (*SVC*) uses the following parameters:

- Fold 1: ’C’: 187.03077394057769,
  ’gamma’: 0.0075590693563175734, ’kernel’: ’linear’.
- Fold 2: ’C’: 5.4021367639052151,
  ’gamma’: 0.073642766499796633, ’kernel’: ’linear’.
- Fold 3: ’C’: 27.977656747557294,
  ’gamma’: 0.00030390547916044405, ’kernel’: ’rbf’.
- Fold 4: ’C’: 7.4608997236358245,
  ’gamma’: 0.053131270021484184, ’kernel’: ’linear’.
- Fold 5: ’C’: 44.734671864296253,
  ’gamma’: 0.053071473092829752, ’kernel’: ’linear’.
- Fold 6: ’C’: 428.38954209781292,
  ’gamma’: 3.2972659091716129e-05, ’kernel’: ’rbf’.
- Fold 7: ’C’: 0.3738904295727859,
  ’gamma’: 0.31352053822907555, ’kernel’: ’linear’.
- Fold 8: ’C’: 0.58819021731891663,
  ’gamma’: 0.0036469424319549117, ’kernel’: ’linear’.
- Fold 9: ’C’: 235.59503011564226,
  ’gamma’: 0.05404750660551369, ’kernel’: ’linear’.
- Fold 10: ’C’: 66.245436465350053,
  ’gamma’: 0.033959364677904134, ’kernel’: ’linear’.

+*sm* with the random forest classifier (*RF*) uses the following parameters:

- Fold 1: ’max_depth’: 27, ’max_leaf_nodes’: 48, ’min_samples_leaf’: 1, ’min_samples_split’: 8, ’n_estimators’: 295.
- Fold 2: ’max_depth’: 23, ’max_leaf_nodes’: 29, ’min_samples_leaf’: 3, ’min_samples_split’: 19, ’n_estimators’: 289.
- Fold 3: ’max_depth’: 26, ’max_leaf_nodes’: 44, ’min_samples_leaf’: 2, ’min_samples_split’: 12, ’n_estimators’: 115.
- Fold 4: ’max_depth’: 23, ’max_leaf_nodes’: 49, ’min_samples_leaf’: 1, ’min_samples_split’: 11, ’n_estimators’: 23.
- Fold 5: ’max_depth’: 18, ’max_leaf_nodes’: 42, ’min_samples_leaf’: 1, ’min_samples_split’: 13, ’n_estimators’: 264.
- Fold 6: ’max_depth’: 22, ’max_leaf_nodes’: 39, ’min_samples_leaf’: 4, ’min_samples_split’: 10, ’n_estimators’: 351.
- Fold 7: ’max_depth’: 21, ’max_leaf_nodes’: 48, ’min_samples_leaf’: 1, ’min_samples_split’: 7, ’n_estimators’: 258.
- Fold 8: ’max_depth’: 23, ’max_leaf_nodes’: 42, ’min_samples_leaf’: 4, ’min_samples_split’: 9, ’n_estimators’: 127.
- Fold 9: ’max_depth’: 25, ’max_leaf_nodes’: 42, ’min_samples_leaf’: 1, ’min_samples_split’: 12, ’n_estimators’: 328.
- Fold 10: ’max_depth’: 22, ’max_leaf_nodes’: 31, ’min_samples_leaf’: 5, ’min_samples_split’: 11, ’n_estimators’: 81.

## Data Availability

Access to data is subject to approval by the PRIMEGE’s scientific council, which evaluates the relevance of the request for the improvement of medical practices (contact email: David.DARMON@univ-cotedazur.fr).

## Abbreviations

ATC: Anatomical, therapeutic and chemical classification system
BOC: Bag-of-concepts
BOW: Bag-of-words
CCS: Clinical classification software
CRFs: Conditonal random fields
EMR: Electronic medical record
GP: general practitioner
HMMs: Hidden Markov models
ICPC-2: International classification of primary care, 2nd edition
LTC: Long term condition
LR: Logistic regression
MEMMs: Maximum entropy models
NDF-RT: National drug file - reference terminology
OWL: Web Ontology Language
RBF: Radial basis function kernel
RF: Random forests
SKOS: Simple knowledge organization system
SNOMED-CT: Systematized nomenclature of clinical terms
SVC: C-support vector classifier
SVM: Support vector machine
UMLS: Unified medical language system

## Footnotes

1 http://www.nlm.nih.gov/pubs/factsheets/umls.html

2 https://github.com/TeamHG-Memex/sklearn-crfsuite

3 https://docs.scipy.org/doc/scipy/reference/generated/scipy.stats.expon.html

4 https://www.w3.org/TR/owl-overview/

5 https://www.w3.org/TR/skos-reference/

6 International Classification of Primary Care http://bioportal.lirmm.fr/ontologies/CISP-2

7 Anatomical, Therapeutic and Chemical https://bioportal.bioontology.org/ontologies/ATC

8 National Drug File - Reference Terminology https://bioportal.bioontology.org/ontologies/NDF-RT

9 https://evs.nci.nih.gov/ftp1/MED-RT/MED-RTDocumentation.pdf

10 http://dbpedia.org

11 https://en.wikipedia.org/

12 Namespace: http://purl.org/dc/terms/

13 http://dbpedia.org/class/yago/

14 dbpedia-fr:<http://fr.dbpedia.org/resource/>

15 category-fr:<http://fr.dbpedia.org/resource/Catégorie:>

16 https://www.wikidata.org/

17 https://wikimediafoundation.org/

18 https://query.wikidata.org/sparql

19 https://docs.scipy.org/doc/scipy/reference/generated/scipy.spatial.distance.correlation.html

20 https://project.inria.fr/corese/

21 https://github.com/kermitt2/entity-fishing

22 https://gate.ac.uk/
